# Predicting and Optimizing Microswimmer Performance from the Hydrodynamics of Its Components: The Relevance of Interactions

**DOI:** 10.1089/soro.2017.0099

**Published:** 2018-08-01

**Authors:** Nicola Giuliani, Luca Heltai, Antonio DeSimone

**Affiliations:** MathLab, SISSA—International School for Advanced Studies, Trieste, Italy.

**Keywords:** micro-swimmers, hydrodynamic interactions, performance optimization, Resistive Force Theory

## Abstract

Interest in the design of bioinspired robotic microswimmers is growing rapidly, motivated by the spectacular capabilities of their unicellular biological templates. Predicting the swimming speed and efficiency of such devices in a reliable way is essential for their rational design, and to optimize their performance. The hydrodynamic simulations needed for this purpose are demanding and simplified models that neglect nonlocal hydrodynamic interactions (e.g., resistive force theory for slender, filament-like objects that are the typical propulsive apparatus for unicellular swimmers) are commonly used. We show through a detailed case study of a model robotic system consisting of a spherical head powered by a rotating helical flagellum that (a) the errors one makes in the prediction of swimming speed and efficiency by neglecting hydrodynamic interactions are never quite acceptable and (b) there are simple ways to correct the predictions of the simplified theories to make them more accurate. We also formulate optimal design problems for the length of the helical flagellum giving maximal energetic efficiency, maximal distance traveled per motor turn, or maximal distance traveled per unit of work expended, and exhibit optimal solutions.

## Introduction and Objectives

The swimming behavior of microscopic organisms is attracting increasing interest, and the literature on this subject is growing at a fast pace. The topic is biologically relevant: the study of motile pathogens and sperm cells can offer new insight in the prevention and treatment of certain diseases^1^ or reproductive disorders.^2^ Many microorganisms may change their swimming behavior depending on the fluid properties,^3^ or near particular interfaces,^4,5^ affecting the functioning of wastewater treatment systems or the contamination of water reservoirs. In addition, motile cells provide a template for the bioinspired design of micrometer-scale, self-sufficient machines capable of executing controlled motion^6,7^ that one may hope to use in biomedical applications. Predicting their behavior when they are immersed in a fluid opens the way to the rational design and performance optimization of artificial robotic microswimmers.^8–13^

As research moves from conceptual principles and proofs of concept to the actual design of bioinspired microrobots, the need for reliable tools to make quantitatively accurate predictions is becoming urgent. The seminal articles^14,15^ by Purcell represent a crucial reading for anyone entering the field. In this study, the author poses the fundamental design problem: given the hydrodynamic resistance properties of a body and a propeller (two matrices), estimate the swimming speed of the assembly when a motor imposes a relative rotation between the two. The author solves the problem by restricting attention to the case where the hydrodynamic resistance of the assembly (body plus propeller) can be estimated as the sum of the individual resistances of the components. This is approximately correct, provided that the hydrodynamic interactions between body and propeller can be neglected. By imposing that the total viscous drag and torque on the assembly vanish (self-propulsion), one can calculate the translational and rotational speed resulting from the rotation of the motor. This also opens the way to evaluating the efficiency of a propeller pushing a body, and to formulating and solving optimal design problems.

The approach pioneered by Purcell, and based on the notion of additivity of resistances is still used as a working tool.^7^ However, we believe that there is an urgent need to reconsider the limitations of this approach, which completely neglects the hydrodynamic interactions between the body and the propeller.

Neglecting hydrodynamic interactions may lead to errors in the prediction of swimming performance that are unacceptably large. This was of course known to Purcell, who cautioned the reader to question the validity of his approach when hydrodynamic interactions cannot be neglected. What seems to be less appreciated is that, in fact, for the geometries typically encountered in applications, hydrodynamic interactions *cannot* be neglected.

Similar remarks apply to the use of simplified methods to evaluate the viscous resistance of slender objects, such as filaments, flagella, and helical propellers. Full hydrodynamic simulations are very expensive from a computational point of view. For this reason, approximations with respect to complete hydrodynamic simulations are needed, and Resistive Force Theory (RFT)^16,17^ has often been employed. In this simplified model, drag is local (the viscous force at one point of a swimmer only depends on the velocity of that point) and hydrodynamic interactions are again neglected. The results obtained with RFT can provide interesting insight on the qualitative behavior of swimmers. For this reason, RFT is one of the most common numerical tool in the study of motion of animal and robots at the microscopic scale or in granular media.^18^ However, we believe that the trust in the ability of RFT to capture the swimming performances in full quantitative detail is sometimes excessive among workers in the field. Only recently has this point of view started to emerge.^19^

Our work has two main objectives: (a) to call attention on the errors one can make by neglecting hydrodynamic interactions when trying to predict and optimize the performance of robotic microswimmers and (b) to look for the possibility of correcting the results that can be obtained with simplified methods (in particular, RFT) to make them applicable, at least in certain regimes. We accomplish this by thoroughly revisiting Purcell's work,^15^ studying in detail the case of a bacterium-like model robotic swimmer consisting of a spherical head propelled by a rotating helical flagellum. In addition, we formulate and solve several optimal design problems, namely, to find the length of the helical propeller that maximizes a suitably chosen performance measure (the energetic efficiency, the distance covered in one flagellar turn, or the distance traveled per unit of work expended).

The biological swimmers that inspire our analysis are bacteria such as *E. coli*, whose helical tail consists of a bundle of elastically deformable (hence soft) flagella. Each filament is attached to a rotary motor at its base. The stationary shape attained by the flagellar bundle when the motors rotate is established by the competition of hydrodynamic forces and elastic restoring forces. For simplicity, artificial constructs mimicking these swimming bacteria use very stiff tails, whose (fixed) shapes reproduce the stationary shapes exhibited by the flagellar bundle. The issue we address in this article, namely, the need to assess the relevance of hydrodynamic interactions in predicting and optimizing the performance of microrobots resulting from the assembly of different body parts is common to both the cases, in which the individual components are deformable, and when each of them is rigid. In the case of deformable components, additional care needs to be taken in the selection of a “nominal” body-fixed reference frame and the reader is referred to Dal Maso *et al*.^20^ for one possible procedure.

Moreover, RFT has been used as a tool to model the motion of deformable locomotors, both animals and robots, which propel themselves in sand, thanks to periodic shape changes.^18^ The issue we raise in this article, namely, of whether the behavior of a system consisting of the assembly of several components can be predicted from the knowledge of the hydrodynamic resistance of the individual components, is an interesting one also in this context, but it has not yet been addressed.

The rest of the article is organized as follows. We consider swimmers in Stokes flow, and use a Boundary Element Method (BEM) solver to simulate the hydrodynamic behavior of a model robotic swimmer. We validate the model using different numerical benchmarks involving both the computation of resistance matrices^19,21^ and the complete simulation of bacterium-like swimmers.^5,22,23^ We then study test cases involving hydrodynamic interactions between different bodies^24^ as benchmarks for the accuracy of our solver (and of its ability to correctly resolve hydrodynamic interactions). We use the insight gained with the analysis of the benchmark problems to predict and optimize the performance of a bacterium-like model robotic swimmer consisting of a spherical head propelled by a rotating helical flagellum.

## Materials and Methods

### Solution of the swimming problem by BEM

We introduce the mathematical formulation of the swimming problem together with the numerical methodology we apply in this work.

#### The swimming problem

Following Refs.^20,25^, a swimmer is a time-dependent bounded open set ${B_t} \in { \mathbb{R}^d}$ with $d = 2 , 3$. The map $\chi :{ \bar B_0} \subset { \mathbb{R}^d} \times [ 0 , T ] \to { \mathbb{R}^d}$ describes the position *x* at time *t* of a material point X, namely,

\begin{align*}
x ( X , t ) = \chi ( X , t ) = q ( t ) + R ( t ) s ( X , t ) , \tag{1}
\end{align*}

where $q ( t )$ represents the translation of a point (origin of the body frame), *R*(*t*) is a rotation tensor describing the rotation of the body frame, and *s*(*X*, *t*) represents the current shape (i.e., the position of all points with respect to the body frame). We set ${B_t} = \chi ( {B_0} , t )$. Using (1), the velocity of any point of the swimmer, and in particular of its boundary $\Gamma = \partial {B_t}$, is given by

\begin{align*}
\begin{split} { u_s } &= { \dot { x } = { \frac { \partial \chi (
X , t ) }  { \partial t } } } \\ & = { { \frac { dq }  { dt } } +
R ( t ) { \frac { \partial s ( X , t ) }  { \partial t } } + {
\frac { dR ( t ) }  { dt } } s ( X , t ) = } \\& = { \dot q ( t )
+ R ( t ) \dot s ( X , t ) + \omega ( t ) \wedge ( R ( t ) s ( X ,
t ) ) . } \end{split} \tag { 2 }
\end{align*}

We assume *s*(*X*, *t*) to be known. The unknowns are *q*(*t*) and *R*(*t*), which, through their time derivatives, determine the linear and angular velocities $\dot q ( t ) , \omega ( t )$. We group the summands of (2) in two parts representing velocities due to rigid movements and shape changes respectively, namely,

\begin{align*}
{u_s} = {u_r} ( X , t ) + v ( X , t ) \tag{3}
\end{align*}

where

\begin{align*}
v ( X , t ) = R ( t )\, \dot{ s} ( X , t ) , \tag{4}
\end{align*}

and we emphasize that *v*(*X*, *t*) is known only if the actual configuration of the swimmer is known. To express ${u_r} ( X , t )$, we need a set of basis functions to represent the rigid velocities of the swimmer (linear and angular velocities). We rewrite (3) as

\begin{align*}
\begin{split}{{u_s}} & { = \dot {  q} ( t ) + \omega ( t ) \wedge
R ( t ) s ( X , t ) + v ( X , t ) }\\ & { = \mathop \sum
\limits_{i = 1}^{{N_r}} {p_i} ( X , t ) {{ \dot p}_i} ( t ) + v (
X , t ) } \\ & { = P ( X , t ) \dot p ( t ) + v ( X , t ) , }
\hfill \end{split}
 \tag{5}
\end{align*}

where ${N_r} = d + {N_ \omega }$ (${N_r} = 3$ if $d = 2$ and ${N_r} = 6$ if $d = 3$), and ${\dot { p}_i} ( t ) = { \dot { q}_i} ( t )$ if $i < d$ and ${ \dot p_i} ( t ) = { \omega _{i - d}} ( t )$ otherwise. We remark that we use the $. \dot ..$ notation in the vector $\dot p ( t )$ even if it does not strictly represent a time derivative, since it consists of both rigid linear and angular velocities (which are not directly the derivatives of $R ( t )$).

We consider self-propelled microswimmers, meaning that no external forces or torques are acting on the system besides those due to viscous drag. Thus, the following system of equation must be fulfilled

\begin{align*}
\int_ \Gamma f ( x ) d \gamma ( x ) = 0 , \tag{6{\rm a}}
\end{align*}

\begin{align*}
\int_ \Gamma f ( x ) \wedge ( x - {x_0} ) d \gamma ( x ) = 0 , \tag{6{\rm b}}
\end{align*}

where *x_0_* is a point of the swimmer (e.g., its center of mass). We remark that if external forces or torques (e.g., gravitational or electromagnetic effects) are present they would only affect the right-hand side of (6), leaving the rest of our methodology unaffected. The distributed forces *f* acting on the surface of the body are given by the action of the Cauchy stress tensor $\sigma$,^26^ namely

\begin{align*}
f = \sigma ( u , p ) n , \tag{7}
\end{align*}

where *n* indicates the outer unit normal vector to the surface, and *u* and *p* represent the velocity and the pressure in the fluid. Since the total external force acting on the swimmer vanishes, the choice of point *x_0_* in Equation (6b) is arbitrary; this would no longer be true in the presence of external forces in the right-hand side of (6a). The resolution of (6) provides the velocities $\dot{  q} ( t ) , \omega ( t )$. Then, their integration in time gives the rigid displacement characterized by $q ( t ) , R ( t )$.

#### Boundary integral equation

**FIG. 1.**
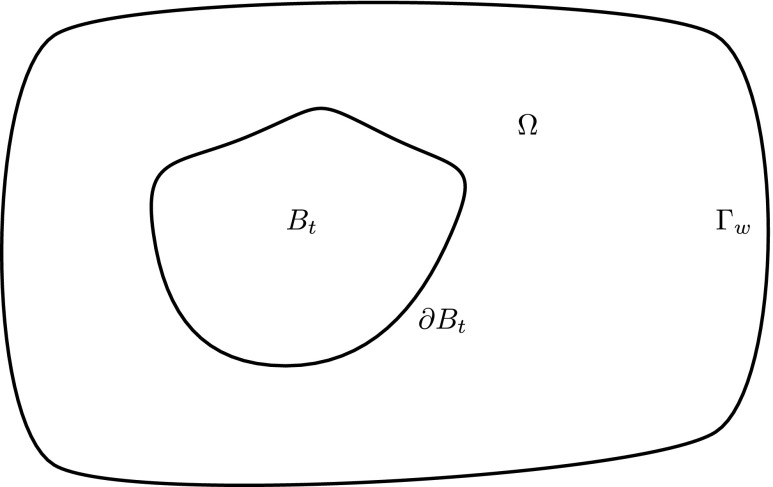
General sketch of the flow domain $\Omega$ with $\partial \Omega = \Gamma$. For a free space swimmer, $\Gamma = \partial {B_t}$ and $\Omega = { \mathbb{R}^d} \backslash \,{\bar { B}_t}$. For a swimmer near no-slip walls, $\Gamma = \partial {B_t} \cup { \Gamma _w}$.

We solve the flow problem in an open set $\Omega$ containing *B_t_* with Lipschitz boundary $\Gamma = \partial \Omega .$. We address both swimming in free space and near physical no-slip interfaces. For a swimmer in free space, $\Gamma = \partial {B_t}$ and $\Omega = { \mathbb{R}^d} \backslash { \bar B_t}$. If no slip walls are present, we denote them as ${ \Gamma _w}$ and consequently, $\Gamma = \partial {B_t} \cup { \Gamma _w}$. We sketch the flow domain in Figure 1.

The Stokes system effectively models the flow around the swimmer,^4,14,15^ and we follow^27,28^ to retrieve a Boundary Integral Formulation. The full representation formula for the velocity is

\begin{align*}
\begin{split}{u_i} ( x ) - \int_ \Gamma {W_{ijk}} ( x , y ) {n_k}
( y ) {u_j} ( y ) d{ \gamma _y} \\\quad= \int_ \Gamma {G_{ij}} ( x
, y ) {f_j} ( y ) d{ \gamma _y} \; \forall x \in { \mathbb{R}^d}
\backslash \Gamma ,\end{split}
 \tag{8}
\end{align*}

where $G , W$ represent the first two Green tensors associated with the fundamental solution of the Stokes system. We consider the trace of (8) to compute the real Boundary Integral Equation (BIE) of the Stokes system

\begin{align*}
\begin{split}&\alpha ( x ) {u_i} ( x ) - \int_ \Gamma ^{PV}
{W_{ijk}} ( x , y ) {n_k} ( y ) {u_j} ( y ) d{ \gamma _y} \\\quad
&= \int_ \Gamma {G_{ij}} ( x , y ) {f_j} ( y ) d{ \gamma _y} \;
\forall x \in \Gamma ,\end{split}
 \tag{9}
\end{align*}

where the integral on the left is computed in the principal value sense of Cauchy, and $\alpha ( x )$ represents the Cauchy principal value (CPV) of such integral at *x*. Equation (9) consists of two boundary integral operators

\begin{align*}
Hu = \int_ \Gamma ^{PV} {W_{ijk}} ( x , y ) {n_k} ( y ) {u_j} ( y ) d{ \gamma _y} , \tag{10{\rm a}}
\end{align*}

\begin{align*}
Vf = \int_ \Gamma ^{PV} {G_{ij}} ( x , y ) {f_j} ( y ) d{ \gamma _y} ,  \tag{10{\rm b}}
\end{align*}

where *H* is the double layer operator and *V* the single layer operator. Using (10), the BIE (9) becomes

\begin{align*}
\left[ { \alpha I - H} \right] u = - \left[ K \right] u = - \left[ V \right] f. \tag{11}
\end{align*}

We define the so-called Dirichlet to Neumann Map as

\begin{align*}
T: [ V{ ] ^{ - 1}} [ K ] , \tag{12}
\end{align*}

and we apply (12) to rewrite the stresses as

\begin{align*}
f = \sigma ( u , p ) n = T{u_s} , \tag{13}
\end{align*}

we remark that (13) is not a punctual relation since $f , {u_s}$ are to be intended as functional of the boundary of the domain $\Gamma$, and we refer the reader to Giuliani^29^ for more details. We apply (5) and (13) to rewrite (6) as

\begin{align*}
\int_ \Gamma T \left( {P ( X , t ) \dot p ( t ) + v ( X , t ) } \right) \;d \gamma = 0 , \tag{14{\rm a}}
\end{align*}

\begin{align*}
\int_ \Gamma T \left( {P ( X , t ) \dot p ( t ) + v ( X , t ) } \right) \; \wedge ( x - {x_0} ) \;d \gamma = 0 , \tag{14{\rm b}}
\end{align*}

and using the definition of rigid modes introduced in (5), we can rewrite (14) in a more compact form as

\begin{align*}
\int_ \Gamma {P^T}T \left( {P ( X , t ) \dot p ( t ) + v ( X , t ) } \right) = 0. \tag{15}
\end{align*}

#### Boundary element method

The numerical resolution of a BIE, like (11), leads to a BEM, and several implementations have been proposed in the literature (see, e.g., Alouges *et al*.^30^ and the references cited therein). Our BEM exploits distributed memory parallelism (MPI) together with existing OpenSOURCE High Performance Computing libraries, such as deal.II^31^ and Trilinos,^32^ to take advantage of modern CPU architectures. A graph partitioning tool, METIS,^33^ automatically handles the work balance between different processors. We use standard Lagrangian finite element spaces on $\Gamma$ to define both the geometry and the basis functions for the unknowns (the velocity *u* and the stresses *f*). We provide the possibility of using both continuous and discontinuous approximation for the solution.

We apply a collocation scheme, namely, we replace the continuous functions *u* and *f* with their numerical approximations (using *N* degrees of freedom) and we collocate the BIE on a number of points equal to the number of unknowns. Collocating (9) produces a linear system of *N* equations in *N* unknowns. We also impose the *N_r_* Equation (15) to impose the balance laws of linear and angular momentum. Thus, we assemble a system of $N + {N_r}$ equations in $N + {N_r}$ unknowns, which is solved using a parallel iterative generalized minimal residual (GMRES) solver. We refer the reader to Giuliani *et al.*^34^ for more details.

### Validation against literature benchmarks

Simple rigidly moving objects and model composite swimmers are the benchmarks we use to validate the methodology presented in “Solution of the swimming problem by BEM” section.

#### Helix

From the linearity of the Stokes system, the forces acting on a rigid body depend linearly on its velocities, through the resistance matrix $\mathcal{R}$, namely

\begin{align*}
\mathcal{F} = \mathcal{R}{ \cal U} , \tag{16}
\end{align*}

where $\mathcal{F}$ represents the forces and torques acting on the body and ${ \cal U}$ its linear and angular velocities. For all the considered resistance matrix entries, following Lauga *et al*.,^21^ we use the notation $\mathcal{R}_{ij}^{ \alpha \beta }$, where the superscript is *FU* giving the *ith* force component induced by the *jth* linear velocity, $F \Omega$ describing the *ith* force component generated by the *jth* angular velocity, *LU* representing the *ith* torque component generated by the *jth* linear velocity, or $L \Omega$ giving the *ith* torque component induced by the *jth* angular velocity. We consider a flagellum modeled as a circular helix with amplitude *b*, pitch $\lambda$, overall length *L*, number of turns ${N_ \lambda } = L / \lambda$, and flagellar thickness *r*. We measure the mean values for the coefficients *R* during a stroke, by which we mean a complete rotation of an angle $\phi = 2 \pi$ along the longitudinal axis. Given the symmetries associated with the flagellum rotation in free space, some coefficients vanish. We recover the pattern shown in (17)

**FIG. 2.**
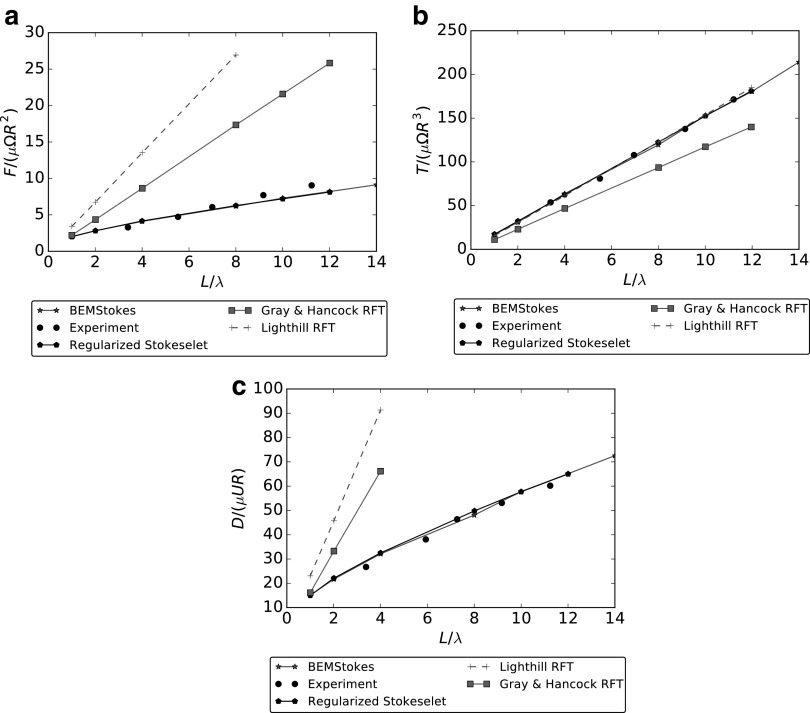
Comparison of the nondimensional resistance coefficients $\mathcal{R}_{xx}^{F \omega } , \mathcal{R}_{xx}^{L \omega } , \mathcal{R}_{xx}^{FU}$, between BEM (*stars*), Gray and Hancock RFT (*squares*), Lighthill RFT (*crosses*), regularized Stokeslet (*pentagons*), and experiment (*circles*). **(a)** Coupling coefficient $\mathcal{R}_{xx}^{F \omega } = F$. **(b)** Torque coefficient $\mathcal{R}_{xx}^{L \omega } = T$. **(c)** Drag coefficient $\mathcal{R}_{xx}^{FU} = D$. BEM, Boundary Element Method; RFT, resistive force theory.

\begin{align*}
{ \mathcal{R}_{spiral}} = \left[ { \begin{matrix} { \mathcal{R}_{xx}^{FU}} & {} & {} & { \mathcal{R}_{xx}^{F \Omega }} & {} & {} \\ {} & { \mathcal{R}_{yy}^{FU}} & {} & {} & { \mathcal{R}_{yy}^{F \Omega }} & { \mathcal{R}_{yz}^{F \Omega }} \\ {} & {} & { \mathcal{R}_{zz}^{FU}} & {} & { \mathcal{R}_{zy}^{F \Omega }} & { \mathcal{R}_{zz}^{F \Omega }} \\ { \mathcal{R}_{xx}^{LU}} & {} & {} & { \mathcal{R}_{xx}^{L \Omega }} & {} & {} \\ {} & { \mathcal{R}_{yy}^{LU}} & { \mathcal{R}_{yz}^{LU}} & {} & { \mathcal{R}_{yy}^{L \Omega }} & {} \\ {} & { \mathcal{R}_{zy}^{LU}} & { \mathcal{R}_{zz}^{LU}} & {} & {} & { \mathcal{R}_{zz}^{L \Omega }} \\ \end{matrix} } \right] , \tag{17}
\end{align*}

and this is consistent with Lauga *et al*.^21^ We follow Rodenborn *et al*.^19^ to perform an analysis of the forces acting on a spiral. We consider $r = b / 16$ and $\lambda = 2.42b$, and we let ${N_ \lambda }$ vary between 1 and 14. The motility of a bacterium in free space is characterized by three main coefficients: $\mathcal{R}_{xx}^{F \Omega } , \mathcal{R}_{xx}^{L \Omega }$, and $\mathcal{R}_{xx}^{FU}$. $\mathcal{R}_{xx}^{F \Omega }$ describes the coupling term expressing the force *F* induced by the spiral rotation, $\mathcal{R}_{xx}^{L \Omega }$ as the reacting torque *T. induced* by the flagellum rotation, and $\mathcal{R}_{xx}^{FU}$ defines the drag *D* due to a translation with unit velocity. We report our comparisons in Figures 2a–2c. We compare, following Rodenborn *et al*.,^19^ our results with RFT,^16,17^ regularized Stokeslet method^35^ and experiments.^19^ Gray and Hancock derived RFT considering that the forces on an infinitesimal segment of a very slender flagellum moving at very low speed can be seen as directly proportional to the velocity of the segment itself and to the viscosity of the fluid (by analogy with those acting on an ellipsoid).^16^ Two different proportionality constants ${C_N} , {C_T}$ acting on normal and tangential velocity, respectively, are introduced

\begin{align*}
 { C_T } = { \frac { 2 \pi \mu }  { \log ( 2q / b ) + 0.5 } } , \tag{18{\rm a}} 
\end{align*}

\begin{align*}
 { C_N } = { \frac { 4 \pi \mu }  { \log ( 2q / b ) - 0.5 } } , \tag{18{\rm b}} 
\end{align*}

which depend on the choice of *q*. Gray and Hancock assumed $q = \lambda$. Lighthill^17^ discussed different models for the coefficients ${C_T} , {C_N}$, both considering a different choice for the parameter ($q = 0.09 \lambda$), and by proposing different expressions replacing (18), which provide a better approximation to the hydrodynamics of slender bodies. In this work, we use the RFT methods considered in Rodenborn *et al*.^19^ and available as “Helical Swimming Simulator” at Matlab File Exchange.^36^

From Figure 2, we see a very good agreement between our method and the expected experimental and numerical results by Rodenborn *et al*.^19^

#### Two spheres

**FIG. 3.**
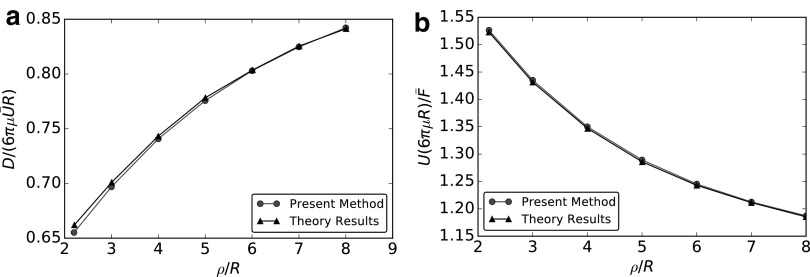
Motility analysis for the two sphere system. **(a)** Drag on a single sphere induced by a system velocity $\bar {  U}$, present results (*circles*), and theoretical benchmark (*triangles*). **(b)** Linear velocity of the two-sphere system induced by an external force $2\, \bar {  F}$, present results (*circles*), and theoretical benchmark (*triangles*).

We analyze the hydrodynamic interactions between two translating spheres of radius *R* separated by a distance $\rho$.^24^ We compute the drag induced on the system by a velocity $\bar {  U}$ parallel to the line joining the centers and the velocity due to an imposed force $2\, \bar {  F}$ directed again along the centerline, and we use the exact solutions by Happel and Brenner^24^ as benchmarks. We let $\rho$ vary from $2.2$ to 8. The decay of the drag as $\rho$ increases (equivalently, the increase in velocity at given force) illustrates the phenomenon of hydrodynamic screening (each sphere moves in the “wake” of the other one) and is due to mutual hydrodynamic interactions between the two spheres. Figure 3a represents the drag induced on one of the spheres by a linear velocity $\bar {  U}$. Circles show the BEM results and triangles represent the theoretical solution. Figure 3b shows the linear velocity induced by an overall force $2\, \bar {  F}$ on the sphere system. We plot with circles the BEM results and with triangles the expected analytical solution. The very good agreement between benchmarks and our results proves that the present methodology properly reproduces the hydrodynamic interactions between two simple moving rigid bodies.

#### Composite swimmer

We now move to a model “bacterium-like” composite system made by a spherical head and a rotating helical flagellum, which has been analyzed in Refs.^4,22,23^ We consider the flagellum as a circular helix with circular cross-section of radius *r* and axis given by the curve

\begin{align*}
{ \bf{r}} = ( x , y , z ) = ( x , bE ( x ) cos ( kx - \omega t ) , bE ( x ) cos ( kx - \omega t ) ) , \tag{19}
\end{align*}

\begin{align*}
E ( x ) = 1 - {e^{ - {{ ( {k_E}x ) }^2}}}. \tag{20}
\end{align*}

**FIG. 4.**
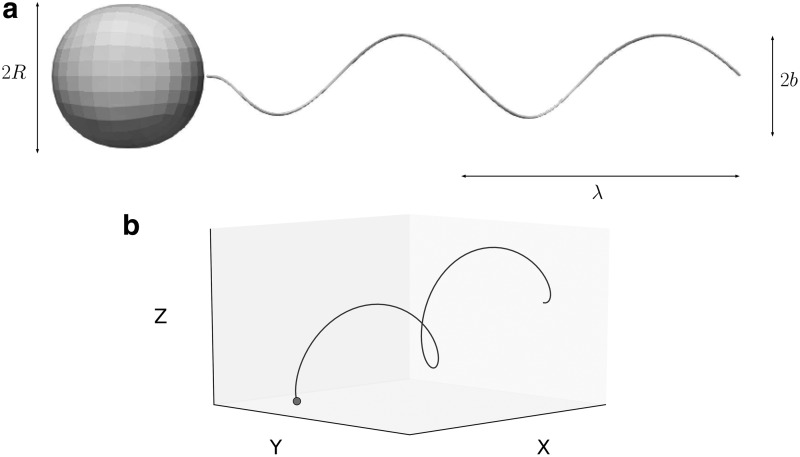
Setting and trajectory for the composite model swimmer. **(a)** Sketch of the geometry for the composite model bacterium composed by a spherical head of radius *R* and a helical flagellum with pitch $\lambda$, and amplitude *b*. **(b)** Three-dimensional trajectory (*line*) of the head-tail juncture starting from the origin (*dot*) for two turns of the helix with respect to the head.

**FIG. 5.**
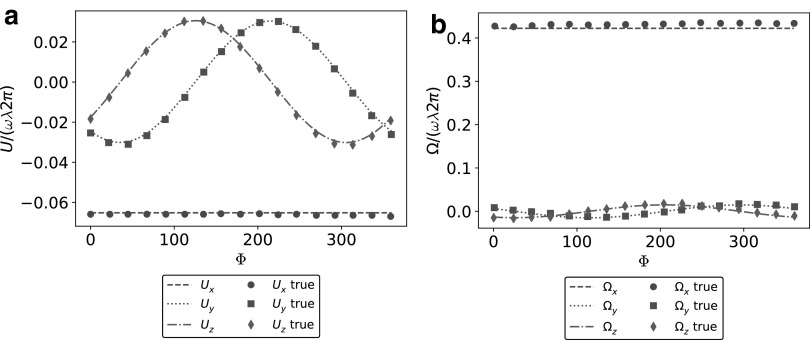
Free space instantaneous velocities. **(a)** Comparison of the linear velocity components along *x-*, *y-*, and *z-*axis between our method (*dashed*, *dotted* and *dashed-dotted lines*) and benchmark results (*dots*, *squares*, and *diamonds*) by Ramia *et al.*^23^ **(b)** Comparison of the angular velocity components along *x-*, *y-*, and *z*-axis between our method (*dashed*, *dotted* and *dashed-dotted lines*) and benchmark results (*dots*, *squares*, and *diamonds*) by Ramia *et al.*^23^

The parameter *k_E_* controls how quickly the helix grows to its steady amplitude *b*, starting from the attachment to the bacterium head. We assume the pitch of the helix to be $\lambda = 2 \pi / k$ and the amplitude to be $b = 1 / k$; the flagellum has ${N_ \lambda } = L / \lambda$ turns. Figure 4 shows the shape of the swimmer, and the three-dimensional trajectory produced by the relative rotation of the helical flagellum with respect to the head (the emergence of helical trajectories of microswimmers is discussed at length in Rossi *et al*.^37^). In real bacteria, a motor on the head provides a torque on the flagellum, which is a deformable continuum, and hydrodynamic interactions induce some stresses on the flagellum boundary that deforms accordingly to counter-balance the imposed torque. At equilibrium, the flagellum assumes a spiral shape as the one depicted in (19). Therefore, after a brief transient, the flagellum rigidly rotates with respect to the head with angular velocity $\omega$ (directed along the *x*-axis). As a consequence, the composite system moves with velocity **V** and angular velocity Ω. To assess the performance of our methodology, we compare the instantaneous linear and angular velocities with the results of Ramia *et al.*^23^ We consider the following set of parameters: $R = 1$, ${N_ \lambda } = 1.5$, $L / R = 10$, and $k = {k_e} = 1 / b$. Figure 5a reports the comparison for the linear velocities, while in Figure 5b, we represent the angular velocities. We analyze the results over a complete revolution of the tail with respect to the head, and we depict the relative rotation with the angle $\Psi$. The continuous lines show our numerical results, while the dots describe the reference solution by Ramia *et al*.,^23^ with different shapes representing different velocity components. The good agreement between our numerical results and the benchmarks^23^ proves that we recover correctly the rigid velocities of a composite swimmer in free space.

To remove some of the symmetries of the free space case, and to have a more stringent test of the accuracy of our numerical method, we study the instantaneous linear velocities of a bacterium near a no-slip wall, as in Ramia *et al*.^23^ We restrict attention to the case where the minimum distance *s_d_* between the wall and the bacterium is

**FIG. 6.**
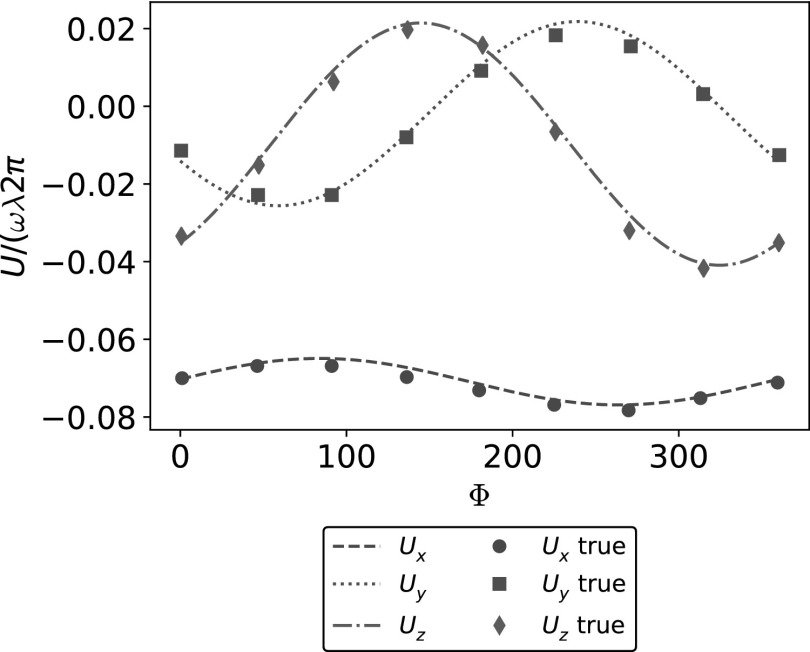
Instantaneous velocity near a no-slip wall. Comparison between our method (*dashed*, *dotted* and *dashed-dotted lines*) and the reference results (*dots*, *squares*, and *diamonds*) by Ramia *et al*.^23^

\begin{align*}
{s_d} = 0.1R , \tag{21}
\end{align*}

where *R* is the radius of the bacterium head. We compare our numerical results (continuous lines) with those by Ramia *et al.*^23^ (dots) in Figure 6, and we see a very good agreement, for all the velocity components (different shapes).

Figure 6 proves that our methodology recovers hydrodynamic interactions both between different parts of a single composite swimmer and between distinct bodies (swimmer and no-slip wall).

## Results: Head-Tail Interactions in a Model Robotic Bacterium

We now move to the study of the impact of hydrodynamic interactions on the performance prediction of a model swimmer made by assembling distinct parts: a “body” and a “propeller.” As a test case, we consider a “robotic” bacterium composed of a rigid head and a rotating helical flagellum with fixed shape. The head is a sphere of radius *R*, and the flagellum is a circular helix such as the one presented in “Validation against literature benchmarks” section, so that we can take advantage of analysis and data presented in Rodenborn *et al*.^19^ By varying the length of the flagellum at fixed head size, we study the significance of hydrodynamic interactions between head and flagellum. We follow Refs.^14,15^ where the author studied the motion of the composite system (head and flagellum), trying to infer its performance from the knowledge of the hydrodynamics of the separate components (body and propeller). We call such methodology the “additive approximation” or “additive approach.” In recent years, “robotic” bacterium models have been used to study the efficiency of microswimming strategies and, consequently, to optimize swimming performances (see Refs.^11–13,38^ for further details). In Raz and Leshansky,^38^ we can find a first discussion on Purcell's additive approximation; however, we believe that a deeper analysis is necessary to understand the limitations of such an approach.

### Additive versus global approach for linear and angular velocity

**FIG. 7.**
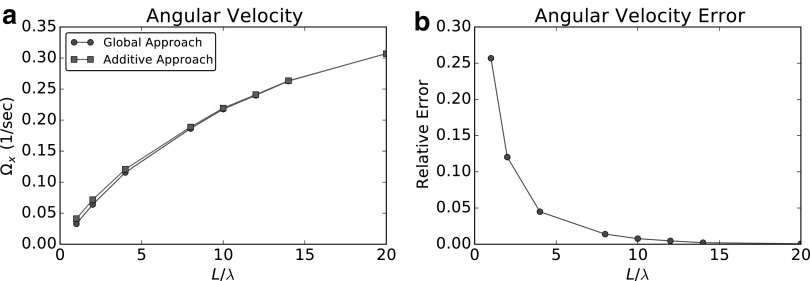
Angular velocity comparison. **(a)** Angular velocity obtained using the global approach (*circles*), and solution obtained with the additive approximation, which neglects the interactions between body and flagellum (*squares*). **(b)** Relative error introduced by the additive approach.

**FIG. 8.**
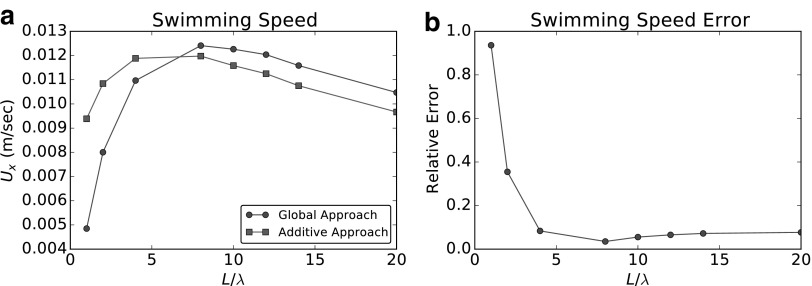
Swimming speed comparison. **(a)** Swimming speed obtained using the global approach (*circles*), and solution obtained with the additive approximation, which neglects the interactions between body and flagellum (*squares*). **(b)** Relative error introduced by the additive approach.

We compare the results obtained using the additive approach (AA) with the accurate resolution, by BEM, of the hydrodynamics of the entire robotic bacterium. We call the latter “global approach” (GA). It is expected from Purcell^15^ that, as the length of the flagellum increases, the error induced by neglecting head-tail interactions should decrease. Thus, we let the number of turns ${N_ \lambda }$ vary from 1 to 20, keeping $\lambda$ and $\omega$ fixed, and we compare both the angular velocity $\Omega$ and the swimming speed *U* obtained with the two approaches. In Figure 7, we report the comparison between AA and GA for the angular velocity $\Omega$: circles in Figure 7a show the solution obtained with the GA, while squares show the results obtained with the AA. Figure 7b represents the relative error introduced by AA. In Figure 8, we compare the results for the swimming speed *U*. Circles in Figure 8a show the GA results and squares are relative to the solution obtained with AA. In Figure 8b, we plot instead the relative error. We see that, for what concerns the angular velocity, the additive approximation does not introduce significant errors. Moreover, such errors decrease quickly as the relative length of the flagellum increases with respect to the head size. However, for what concerns the swimming speed, the error is never negligible for any of the configurations considered: it is very significant for short flagella (small ${N_ \lambda }$), and it stabilizes at a relatively small value (∼10%) for ${N_ \lambda } > 5$. While both approaches lead to a maximum in the velocity, these maxima are observed for different values of ${N_ \lambda }$.

### A simple formula provides a correction for the AA

We want to understand the differences in swimming speed *U* computed using either the additive approximation or the GA for the entire bacterium. In the additive approximation, we can write, following Purcell,^15^ the momentum balance laws for the swimmer as

\begin{align*}
\left[ { \begin{matrix} {{A_0}} & {} \\ {} & {{C_0}} \\ \end{matrix} } \right] \left[ { \begin{matrix} U \\ \Omega \\ \end{matrix} } \right] + \left[ { \begin{matrix} A & B \\ B & C \\ \end{matrix} } \right] \left[ { \begin{matrix} U \\ { \Omega - \omega } \\ \end{matrix} } \right] = 0 , \tag{22}
\end{align*}

where ${A_0} , {C_0}$ represent the drag and torque coefficients for the head, $A , B , C$ are the drag, coupling, and torque coefficients for the propeller, and $U , \Omega$ are the unknown linear and angular velocities of the head, while $\omega = { \Omega _{head}} - { \Omega _{flagellum}}$ represents the prescribed relative angular velocity of the helical flagellum with respect to the head. Solving for *U* and $\Omega$, we obtain

\begin{align*}
U = { \frac { { C_0 } B }  { ( { C_0 } + C ) ( { A_0 } + A ) - { B^2 } } } \omega , \tag{23{\rm a}} 
\end{align*}

\begin{align*}
\Omega = \left( { 1 - { \frac { ( { A_0 } + A ) { C_0 } }  { ( { C_0 } + C ) ( { A_0 } + A ) - { B^2 } } } } \right) \omega . \tag{23{\rm b}} 
\end{align*}

If we consider the hydrodynamics of the entire system, without invoking the additive approximation, we write the velocity field of the swimmer as

\begin{align*}
\begin{split}\underline v ( x ) = & {U_1} \underline e { \chi _1} (
x ) + {U_2} \underline e { \chi _2} ( x ) + { \Omega _1}
\underline e \wedge ( x - {x_o} ) { \chi _1} ( x ) \\\quad & + {
\Omega _2} \underline e \wedge ( x - {x_o} ) { \chi _2} ( x )
,\end{split}
 \tag{24}
\end{align*}

where ${ \chi _1}$ and ${ \chi _2}$ are the characteristic functions of body 1 (the head) and 2 (the propeller), that is, ${ \chi _1} ( x ) = 1$ if *x* belongs to body 1 and ${ \chi _1} ( x ) = 0$ otherwise. Moreover, $\underline e$ is a unit vector along the axis of the helical flagellum. Using the linearity of the Stokes system, we can write the force and torque (with respect to the pole *x_o_*) acting on the whole body, respectively, as

\begin{align*}
{A_1}{U_1} + { \hat {  B}_1}{ \Omega _1} + {A_2}{U_2} + { \hat {  B}_2}{ \Omega _2} , \tag{25{\rm a}}
\end{align*}

\begin{align*}
{ \bar {  B}_1}{U_1} + {C_1}{ \Omega _1} + { \bar {  B}_2}{U_2} + {C_2}{ \Omega _2} , \tag {25{\rm b}}
\end{align*}

where *A_1_* is the viscous force on the whole system arising from the velocity field (24) with ${U_1} = 1$ and ${U_2} = { \Omega _1} = { \Omega _2} = 0$. A similar interpretation holds for the other coefficients ${ \hat { B}_i}$ (giving forces induced by rotation of the body *i*, in the presence of the other body parts kept fixed), ${ \bar B_i}$ (giving torques induced by translation of the body *i*, in the presence of the other body parts kept fixed), and *C_i_* (giving torques induced by rotation of the body *i*, in the presence of the other body parts kept fixed). Writing *U* for *U_1_* and $\Omega$ for ${ \Omega _1}$, requiring that

\begin{align*}
{U_2} = U , \tag{26{\rm a}}
\end{align*}

\begin{align*}
{ \Omega _2} = \Omega - \omega , \tag{26{\rm b}}
\end{align*}

and that the total viscous forces and torques (25) vanish, we obtain the system

\begin{align*}
\left[ { \begin{matrix} {{A_1}} & {{{ \hat { B}}_1}} \\ {{{ \bar B}_1}} & {{C_1}} \\ \end{matrix} } \right] \left[ { \begin{matrix} U \\ \Omega \\ \end{matrix} } \right] + \left[ { \begin{matrix} {{A_2}} & {{{ \hat { B}}_2}} \\ {{{ \bar B}_2}} & {{C_2}} \\ \end{matrix} } \right] \left[ { \begin{matrix} U \\ { \Omega - \omega } \\ \end{matrix} } \right] = 0. \tag{27}
\end{align*}

Notice that we can rewrite (27) as

\begin{align*}
\left[ { \begin{matrix} \mathcal{R} \\ \end{matrix} } \right] \left[ { \begin{matrix} U \\ \Omega \\ \end{matrix} } \right] = \left[ { \begin{matrix} {{A_2}} & {{{ \hat B}_2}} \\ {{{ \bar B}_2}} & {{C_2}} \\ \end{matrix} } \right] \left[ { \begin{matrix} 0 \\ \omega \\ \end{matrix} } \right] , \tag{28}
\end{align*}

where

\begin{align*}
\left[ { \begin{matrix} \mathcal{R} \\ \end{matrix} } \right] = \left[ { [ { \mathcal{R}_1} + { \mathcal{R}_2} ] } \right] = \left[ { \left[ { \begin{matrix} {{A_1}} & {{{ \hat { B}}_1}} \\ {{{ \bar B}_1}} & {{C_1}} \\ \end{matrix} } \right] + \left[ { \begin{matrix} {{A_2}} & {{{ \hat { B}}_2}} \\ {{{ \bar B}_2}} & {{C_2}} \\ \end{matrix} } \right] } \right] \tag{29}
\end{align*}

is the resistance matrix of the complete swimmer. Thus, $\mathcal{R}$ is symmetric (by reciprocity) and positive definite,^24^ even though the two summands ${ \mathcal{R}_1}$ and ${ \mathcal{R}_2}$ defining $\mathcal{R}$ are not individually symmetric. Therefore ${ \hat { B}_1} + { \hat { B}_2} = { \bar B_1} + { \bar B_2}$ (and we will write ${B_1} + {B_2}$ for any of these two sums) and *R* is invertible. Solving for *U* and $\Omega$, we obtain

\begin{align*}
U = { \frac { ( { B_1 } + { B_2 } ) \left( { ( { C_1 } + { C_2 } ) { \frac { { { \hat { B } } _2 } }  { { B_1 } + { B_2 } } } - { C_2 } } \right) }  { ( { C_1 } + { C_2 } ) ( { A_1 } + { A_2 } ) - { { ( { B_1 } + { B_2 } ) } ^2 } } } \omega , \tag{30{\rm a}} 
\end{align*}

\begin{align*}
\Omega = \left( { { \frac { { { \hat { B } } _2 } }  { { B_1 } + { B_2 } } } - { \frac { ( { A_1 } + { A_2 } ) \left( { ( { C_1 } + { C_2 } ) { \frac { { { \hat { B } } _2 } }  { { B_1 } + { B_2 } } } - { C_2 } } \right) }  { ( { C_1 } + { C_2 } ) ( { A_1 } + { A_2 } ) - { { ( { B_1 } + { B_2 } ) } ^2 } } } } \right) \omega . \tag{30{\rm b}} 
\end{align*}

**FIG. 9.**
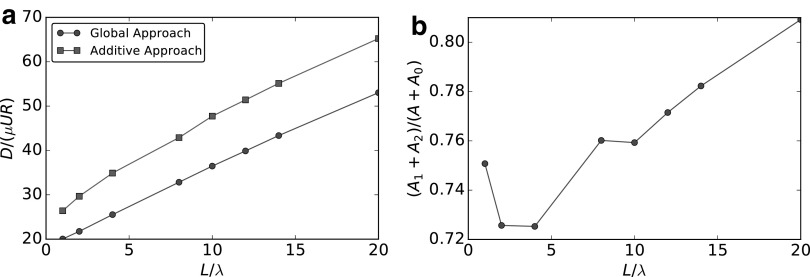
Comparison of the axial (along *x*) drag induced by a longitudinal (along *x*) swimming speed. **(a)** Comparison between the coefficient obtained with the global approach (*circles*) and sum of body and flagellum coefficients computed separately using the additive approach (*squares*). **(b)** Ratio between the global drag and the sum of the two separate contributions of head and flagellum.

**FIG. 10.**
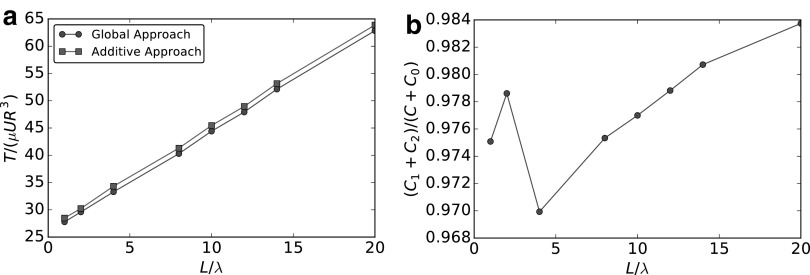
Comparison of the axial (along *x*) torque induced by a longitudinal (along *x*) angular velocity. **(a)** Comparison between the coefficient obtained with the global approach (*circles*) and sum of body and flagellum coefficients computed separately using the additive approach (*squares*). **(b)** Ratio between the global drag and the sum of the two separate contributions of head and flagellum.

Our analysis is focused on a robotic bacterium-like problem, but we remark that for any composite swimmer it is possible to compare the rigid velocities obtained using AA and GA to find the discrepancies and, eventually, even a corrective factor of AA. We analyze the differences between (23a) and (30a) to understand the discrepancies in Figure 8. In the AA, ${A_0} + A$ and ${C_0} + C$ represent the global hydrodynamic coefficients for drag and torque experienced by the whole swimmer, and the single elements ${A_0} , A , {C_0} , C$ are coefficients for drag and torque experienced by swimmer parts considered alone in free space. In the GA, the global hydrodynamic coefficients are given by ${A_1} + {A_2}$ and ${C_1} + {C_2} ,$ where the single components ${A_1} , {A_2} , {C_1} , {C_2}$ represent, as already mentioned, the drag and torque experienced by the whole swimmer induced by the movement of one of its parts, computed considering the presence of all the other parts kept fixed. In Figure 9a, we compare the drag coefficient of the composite system (circles) with the sum of the drags of the single components, namely, body and flagellum, computed separately (squares). In Figure 9b, we plot the ratio between these two quantities. Figure 10a compares the complete torque coefficient in the GA (circles) with the sum of the coefficients of head and propeller computed using the additive approximation (squares). In Figure 10b, we plot the ratio between these two quantities.

As expected from hydrodynamic screening (“Two spheres” section), which is only present in the GA, the additive approximation always overestimates both drag and torque coefficients. From Figures 10b and 9b, we see that if $L \le 10 \lambda$, the screening effect is not fully developed, and this causes the nonregular behavior of the two curves. The rationale behind the fact that AA gives poor results for the drag due to translations and good ones for torque due to rotations is the following: a translating sphere can be modeled as a Stokeslet, with a slow (linear) decay of the induced velocity as the distance from the source increases, and the flagellum is never far enough to neglect hydrodynamic interactions. By contrast, a rotating sphere can be described as a rotlet, with a faster (quadratic) decay. Hydrodynamic interactions between head and propeller are weaker in this case, and the AA safely estimates the overall coefficient. For a detailed analysis of the convergence of the resistance coefficients computed in the GA to the ones obtained with the additive approximation, the reader is referred to Giuliani.^29^

**FIG. 11.**
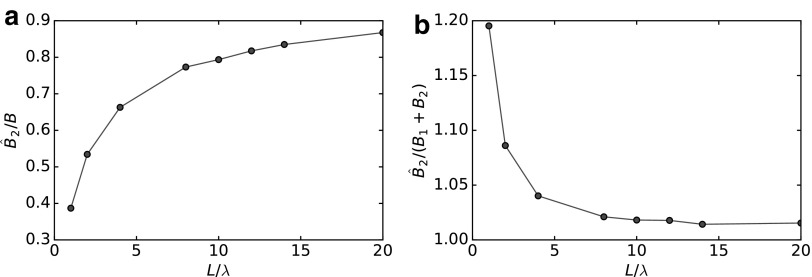
Comparison of the coupling coefficient representing the axial (along *x*) force induced by a longitudinal (along *x*) angular velocity. **(a)** Ratio between the coupling coefficient due to the flagellum in the global approach and the coupling coefficient of the flagellum in free space. **(b)** Ratio between the coupling coefficient due to the flagellum and the complete coupling coefficient, both computed using the global approach.

The swimming speed *U* is also influenced by the coupling coefficients, which are different using the two approaches. We notice that when ${A_0} + A \sim {A_1} + {A_2}$ (only approximately satisfied when the flagellum is very long compared to the head size, $L / \lambda > 15$) and ${C_0} + C \sim {C_1} + {C_2}$ (always true), (30a) collapses into (23a) when ${\, \hat { B}_2} / B \sim 1 ,\, { \hat { B}_2} / ( {B_1} + {B_2} ) \sim 1$. We study these last two conditions in Figure 11. Figure 11a shows the ratio ${\, \hat { B}_2} / B$ and highlights the influence of the head on the flagellum coupling coefficient. In Figure 11b, we plot ${\, \hat { B}_2} / ( {B_1} + {B_2} )$ that represents the relative importance of the flagellum coupling coefficient in the GA. We note that the head contribution represents a minor part of the total coupling coefficient already when ${N_ \lambda } > 2$. Thus we can safely neglect this contribution considering ${\, \hat { B}_2} \sim ( {B_1} + {B_2} )$. However, from Figure 11a, we see that ${\, \hat { B}_2} \sim B$ is approximately satisfied only when the flagellum is very long ($L / \lambda > 15$) compared to the head size, meaning that the flagellum coupling coefficient is strongly influenced by the presence of the spherical head.

Summarizing, because of the screening effect induced by the translating spherical head, we have, ${A_1} + {A_2} \nsim {A_0} + A$. Moreover, the head-tail hydrodynamic interactions cause ${\, \hat { B}_2} \nsim B$, as revealed by the GA. These two conditions provide an explanation for the error introduced by the AA, shown in Figure 8a.

A combination of the resistance coefficients introduced above provides a simple, yet effective correction for the AA. As suggested by the previous analysis, we can consider ${ \hat B_2} \sim ( {B_1} + {B_2} )$ and ${C_1} \sim {C_0} , {C_2} \sim C$. Therefore, we can write (30a) as

\begin{align*}
U = { \frac { ( { B_1 } + { B_2 } ) { C_0 } }  { ( C + { C_0 } ) ( { A_1 } + { A_2 } ) - { { ( { B_1 } + { B_2 } ) } ^2 } } } \omega , \tag { 31 } 
\end{align*}

and we notice that ${ \frac { { { ( { B_1 } + { B_2 } ) } ^2 } }  { ( { A_1 } + { A_2 } ) } } \sim 0$ and ${ \frac { { B^2 } }  { ( A + { A_0 } ) } } \sim 0$ getting

\begin{align*}
U = { \frac { ( { B_1 } + { B_2 } ) }  { ( { A_1 } + { A_2 } ) } } { \frac { { C_0 } }  { ( C + { C_0 } ) - { \frac { { { ( { B_1 } + { B_2 } ) } ^2 } }  { ( { A_1 } + { A_2 } ) } } } } \omega \sim { \frac { ( { B_1 } + { B_2 } ) }  { ( { A_1 } + { A_2 } ) } } { \frac { { C_0 } }  { ( C + { C_0 } ) } } \omega . \tag { 32 } 
\end{align*}

Using the same approximations, we can rewrite (23a) as

\begin{align*}
U = { \frac { ( B ) }  { ( A + { A_0 } ) } } { \frac { { C_0 } }  { ( C + { C_0 } ) } } \omega . \tag { 33 } 
\end{align*}

The ratio between Equations (32) and (33) provides a correcting factor $\upsilon$ for the swimming speed *U*. Namely, we write such a correction as

\begin{align*}
\upsilon = { \frac { ( { A_0 } + A ) ( { B_1 } + { B_2 } ) }  { ( { A_1 } + { A_2 } ) B } } , \tag { 34 } 
\end{align*}

and we notice that $\upsilon$ depends only on geometric parameters, that is, with our assumptions,

\begin{align*}
\upsilon = \upsilon \left( { \frac { L }  { \lambda } } \right) . \tag { 35 } 
\end{align*}

Thus, we can write

\begin{align*}
 { U_ { corr } } =\upsilon { U } = \upsilon \left( { \frac { L }  { \lambda } } \right) { \frac { { C_0 } B }  { ( { C } + { C_0 } ) ( { A } + { A_0 } ) - { B^2 } } } . \tag { 36 } 
\end{align*}

**FIG. 12.**
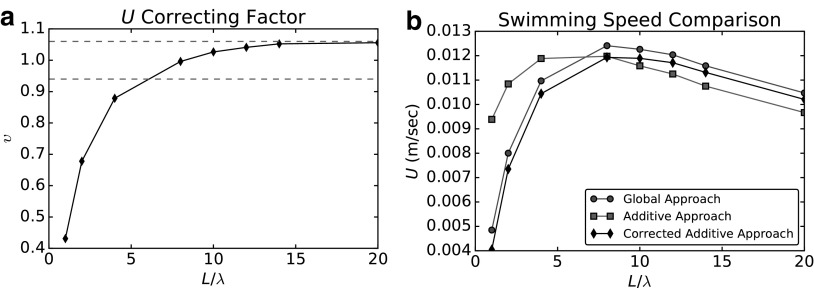
Swimming speed correction for the additive approach. **(a)** Correcting factor $\upsilon$ (*diamonds*), and error thresholds corresponding to $\pm 6 \%$(*dashed lines*). **(b)** Swimming speed obtained with the global approach (*circles*), solution using the additive approximation (*squares*), and the results of the additive approach corrected using $\upsilon$ (*diamonds*).

We study the correcting factor $\upsilon$ in Figure 12. Figure 12a shows $\upsilon$ as a function of flagellum length, while Figure 12b shows a comparison among the predictions of the swimming speed computed using the GA (circles), the AA (squares), and the corrected AA (diamonds). The correcting factor $\upsilon$ greatly improves the accuracy of the prediction of the swimming speed that can be obtained using the additive approximation. Moreover, it is very close (to within less than $6 \%$) to 1 if we consider a sufficiently long tail ($L > 5 \lambda$). This is consistent with Purcell^15^since a system with a long flagellum should be well approximated by considering separately head and tail. In Figure 12a, the dashed lines show the two error thresholds corresponding to $\pm 6 \%$.

The correcting factor $\upsilon$ represents a simple way to improve dramatically the results that can be obtained, considering the additive approximation. Despite its simplicity, the correction recovers the most important features of the hydrodynamic interactions between head and flagellum. In the regime of geometries (${N_ \lambda } < 3 \lambda$ and $\lambda > 6b$), in which it is safe to use RFT to compute the hydrodynamic coefficients of the flagellum,^19^ the correcting factor $\upsilon$ makes it possible to use RFT to safely predict the performance of the swimmer. This is the main result of this article and it is further discussed in the “Correction of RFT predictions” section.

### Correction of RFT predictions

The correcting factor $\upsilon$ is effective even on predictions of the swimming speed *U* based on RFT (Helix Section). To prove this, we consider, as flagellum, a circular helix with the following parameters: $a = b / 16 , \lambda = 8b , {N_ \lambda } = 2$, which is a typical helix that can be well studied using RFT.^19^ We consider again a sphere of radius $R = 2b$ as head and we estimate the error introduced both by the AA and by classical RFT methods. We apply such methodologies following Gray and Hancock^16^ and Lighthill,^17^ and we use the results of the GA as reference. Table 1 shows the comparison between the discussed approaches. We correct the approximated results by AA and RFT using $\upsilon$ computed as follows: we evaluate the coefficients ${A_0} , A , B , C , {C_0}$ using either AA (there the resistance coefficients are evaluated using BEM on the flagellum alone in free space) or RFT (here the resistance coefficients are evaluated using RFT^36^), while we compute $( {A_1} + {A_2} ) , ( {B_1} + {B_2} )$ using BEM on the whole system (head+propeller). The Table proves that $\upsilon$ is not only able to reduce the error of the AA but can also be applied to RFT predictions to reduce the relative errors by at least one order of magnitude.

**Table 1.** Comparison Between Estimates of the Swimming Speed *U* Using Different Approximations

*Model*	U	*Error*
Global approach	0.02301	
Additive approach	0.02512	9.17%
Additive approach corrected	0.02261	1.72%
RFT Gray Hancock	0.02644	14.89%
RFT Gray Hancock corrected	0.02460	1.49%
RFT Lighthill	0.03276	42.37%
RFT Lighthill corrected	0.02284	0.74%

### Efficiency and optimal design

We want to investigate the importance of hydrodynamic interactions in the computation of the efficiency of microswimmers. Several notions of efficiency exist, and we consider here three kinds: energetic efficiency, propulsion efficiency, and swimming efficiency. The first one is the ratio between useful work rate performed by the system and total power input in the system. The second one is the net displacement in one cycle (or stroke), that is, a normalization of the swimming velocity. The third one is the net displacement per unit of work expended.

### Energetic efficiency

The input power is the one expended by the motor and is the product of the torque acting on the flagellum multiplied by the relative angular velocity $\omega$. There are different choices for the useful work rate. Following Refs.^15,22^, one option is the power expended to move the head at velocity *U*, so that

\begin{align*}
 { \eta _ { en , 1G } } = { \frac { { D_ { head } } U }  { { T_ { motor } } \omega } } = { \frac { { A_0 } { U^2 } }  { { T_ { motor } } \omega } } . \tag { 37 } 
\end{align*}

Here ${D_{head}} = {A_0}U$ is the drag that would be experienced by the head moving alone in free space. The head contains the payload of our robotic swimmer, and thus is the only term allowed to contribute to the useful work rate. The other terms in (37), namely *U* and ${T_{motor}}$, need to be computed as functions of the motor angular speed, which is the input datum of the propulsion problem. In (37), these quantities are computed by resolving hydrodynamics in full detail.

Using the additive approximation, we can write (37) as

\begin{align*}
 { \eta _ { en , 1A } } = { \frac { { A_0 } { C_0 } { B^2 } }  { ( ( A + { A_0 } ) C - { B^2 } ) ( ( A + { A_0 } ) ( C + { C_0 } ) - { B^2 } ) } } . \tag { 38 } 
\end{align*}

Another possibility to define energetic efficiency takes into account the drag of the entire bacterium, see Kanehl and Ishikawa,^39^ namely

\begin{align*}
 { \eta _ { en , 2G } } = { \frac { { D_ { total } } U }  { { T_ { motor } } \, \omega } } = { \frac { ( { A_1 } + { A_2 } ) { U^2 } }  { { T_ { motor } } \, \omega } } , \tag { 39 } 
\end{align*}

where $( {A_1} + {A_2} ) U$ is the drag experienced by the whole swimmer, consisting of both the head (the payload) and the flagellum (its propulsive apparatus) when it moves rigidly at speed *U*. Using the additive approximation, this can be expressed as

\begin{align*}
 { \eta _ { en , 2A } } = { \frac { ( A + { A_0 } ) { C_0 } { B^2 } }  { ( ( A + { A_0 } ) C - { B^2 } ) ( ( A + { A_0 } ) ( C + { C_0 } ) - { B^2 } ) } } , \tag { 40 } 
\end{align*}

which is Equation (14) in Purcell.^15^ For the example at hand, we favor ${ \eta _{en , 1}}$ over ${ \eta _{en , 2}}$ as the “correct” notion of energetic efficiency. However, we remark that identifying correctly a notion of “useful work” is not always immediate.

We use the factor $\upsilon$, computed using (34), to correct the energetic efficiencies. In view of (36), and since both (37) and (39) depend quadratically on the swimming velocity *U*, we introduce a corrected AA as

\begin{align*}
 { \eta _ { en , 1 \upsilon } } = { \upsilon ^2 } { \eta _ { en , 1A } } = { \upsilon ^2 } { \frac { { A_0 } { C_0 } { B^2 } }  { ( ( A + { A_0 } ) C - { B^2 } ) ( ( A + { A_0 } ) ( C + { C_0 } ) - { B^2 } ) } } , \tag { 41 } 
\end{align*}

and

\begin{align*}
 { \eta _ { en , 2 \upsilon } } = { \upsilon ^2 } { \eta _ { en , 2A } } = { \upsilon ^2 } { \frac { ( A + { A_0 } ) { C_0 } { B^2 } }  { ( ( A + { A_0 } ) C - { B^2 } ) ( ( A + { A_0 } ) ( C + { C_0 } ) - { B^2 } ) } } . \tag { 42 } 
\end{align*}

**FIG. 13.**
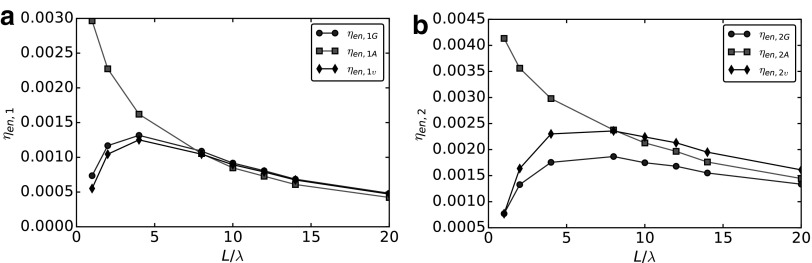
Analysis of the energetic efficiencies. Comparison between global approach (*circles*), additive approximation (*squares*), and corrected additive approach (*diamonds*). **(a)** Energetic efficiency ${ \eta _{en , 1}}$. **(b)** Energetic efficiency ${ \eta _{en , 2}}$.

Figure 13 compares the energetic efficiencies computed using the additive approximation (squares), the GA by BEM (circles), and the corrected AA (diamonds). In Figure 13a, we compare ${ \eta _{en , 1}}$, while in Figure 13b, we analyze ${ \eta _{en , 2}}$. Neglecting head-tail hydrodynamic interactions greatly influences both the energetic efficiencies considered. Figures 13a and b show that, using the GA, a maximum energetic efficiency emerges at intermediate flagellar lengths. This maximum cannot be detected using the additive approximation, while it is recovered using the ${ \upsilon ^2}$ correction.

### Propulsion efficiency

Following Magariyama and Kudo,^3^ a possible measure of the propulsion efficiency is the linear distance covered per flagellar revolution, namely,

\begin{align*}
 { \eta _ { pr , 1G } } = \frac { U }  { { \omega - \Omega } } . \tag { 43 } 
\end{align*}

Using the additive approximation, this becomes

\begin{align*}
 { \eta _ { pr , 1A } } = \frac { B }  { { A + { A_0 } } } . \tag { 44 } 
\end{align*}

Alternatively, it may be useful to consider the distance covered per motor revolution

\begin{align*}
 { \eta _ { pr , 2G } } = \frac { U }  { \omega } , \tag { 45 } 
\end{align*}

which becomes, using the AA,

\begin{align*}
 { \eta _ { pr , 2A } } = { \frac { { C_0 } B }  { ( { C_0 } + C ) ( { A_0 } + A ) - { B^2 } } } . \tag { 46 } 
\end{align*}

Since both (43) and (45) depend linearly on the swimming velocity, we use $\upsilon$ to introduce the corrected swimming efficiencies as

\begin{align*}
 { \eta _ { pr , 1 \upsilon } } = \upsilon { \eta _ { pr , 1A } } = \upsilon \frac { B }  { { A + { A_0 } } } , \tag { 47 } 
\end{align*}

and

\begin{align*}
 { \eta _ { pr , 2 \upsilon } } = \upsilon { \eta _ { pr , 2A } } = \upsilon { \frac { { C_0 } B }  { ( { C_0 } + C ) ( { A_0 } + A ) - { B^2 } } } . \tag { 48 } 
\end{align*}

**FIG. 14.**
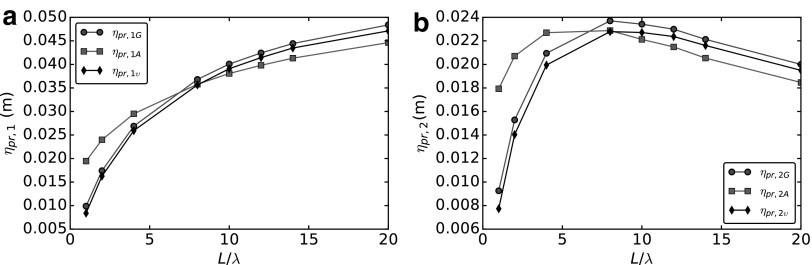
Analysis of the propulsion efficiencies. Comparison between global approach (*circles*), additive approximation (*squares*), and corrected additive approach (*diamonds*). **(a)** Swimming efficiency ${ \eta _{pr , 1}}$. **(b)** Swimming efficiency ${ \eta _{pr , 2}}$.

In Figure 14, we compare the propulsion efficiencies computed using the additive approximation (squares), the GA (circles), and the corrected additive approximation (diamonds). Both propulsion efficiencies are greatly influenced by hydrodynamic interactions. In particular, we see that there exists an optimal value of $L / \lambda$ maximizing ${ \eta _{pr , 2}}$, and this value differs significantly if we consider the global or the AA. The correcting factor $\upsilon$ greatly improves the approximation obtained using the AA. The fact that ${ \eta _{pr , 1}}$ shows a monotonic behavior with respect to tail length, may be due to the fact that, as the tail length increases, $\Omega$ increases. Therefore, since $\omega$ is fixed, the overall flagellar angular velocity $\Omega - \omega$ decreases, and this compensates the decrease of the swimming velocity causing the monotonicity of ${ \eta _{pr , 1}}$.

### Swimming efficiency

The swimming efficiency is defined in Li and Tang^40^ as the translational velocity normalized by the motor torque,

\begin{align*}
 { \eta _ { sw , G } } = \frac { U }  { { { T_ { motor } } \, \omega } } . \tag { 49 } 
\end{align*}

Put differently, ${ \eta _{sw , G}}$ represents the distance traveled per unit of work expended. In the additive approximation, this becomes

\begin{align*}
 { \eta _ { sw , A } } = \frac { B }  { { ( C ( A + { A_0 } ) - { B^2 } )\, \omega } } . \tag { 50 } 
\end{align*}

The inverse of (49) is the work per traveled distance, see Refs.^8,41^, and we introduce this measure of performance as

\begin{align*}
 { w_G } = \frac { { { T_ { motor } } \, \omega } }  { U } . \tag { 51 } 
\end{align*}

Using the additive approximation, (51) reads

\begin{align*}
 { w_A } = \frac { { ( C ( A + { A_0 } ) - { B^2 } ) \omega } }  { B } . \tag { 52 } 
\end{align*}

We use the factor $\upsilon$, computed according to (34), to correct the swimming efficiency and the work per traveled distance that can be obtained using the additive approximation. Considering the linear dependence of (49) on the swimming velocity, we use $\upsilon$ to introduce a corrected swimming efficiency as

\begin{align*}
 { \eta _ { sw , \upsilon } } = \upsilon\, { \eta _ { sw , A } } = \upsilon \frac { B }  { { ( C ( A + { A_0 } ) - { B^2 } } } . \tag { 53 } 
\end{align*}

The work per traveled distance is inversely proportional to the swimming velocity, so we use $1 / \upsilon$ to introduce a corrected work per traveled distance as

\begin{align*}
 { w_ \upsilon } = \frac { 1 }  { \upsilon } { w_A } = \frac { 1 }  { \upsilon } \frac { { ( C ( A + { A_0 } ) - { B^2 } )\, \omega } }  { B } . \tag { 54 } 
\end{align*}

**FIG. 15.**
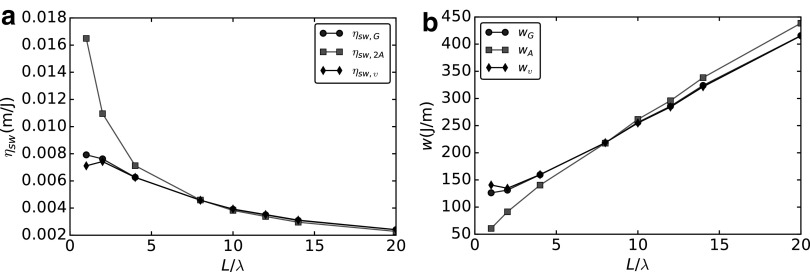
Analysis of the swimming efficiency ${ \eta _{sw}}$ **(a)** and the work per traveled distance *w* **(b)**. Comparison between global approach (*circles*), additive approximation (*squares*), and corrected additive approach (*diamonds*).

In Figure 15, we compare both the swimming efficiency and the work per traveled distance computed using the additive approximation (squares), the GA (circles), and the corrected additive approximation (diamonds). We see that neither the GA nor the AA admits optimal values for ${ \eta _{sw}}$ and *w*; however, we see that the correcting factor $\upsilon$ improves greatly the accuracy of AA.

Neglecting hydrodynamic interactions has an impact both on the swimming efficiency and on the work per traveled distance. However, the correcting factor $\upsilon$ guarantees much better accuracy of the prediction one can make by using the additive approximation.

## Conclusions

In the presence of (nonlocal) hydrodynamic interactions, the possibility of predicting the swimming behavior of an assembly (body plus propeller) from the knowledge of the hydrodynamic properties of its isolated components is conceptually wrong. How can we say anything about the behavior of the assembly if we provide no information on how the presence of the body modifies the flow generated by the rotating propeller? Obviously, Purcell was aware of this fact, and the hypothesis behind his calculations is that the proximity of the body does not appreciably disturb the flow around the propeller, and that this can be a reasonably good approximation if most of the propeller is relatively far from the body (e.g., if the propeller has a helical flagellar shape and the flagellum is sufficiently long).

What we find is that, at least with our geometry (a spherical head attached to a rotating helical tail), this is never quite the case. In other words, the predictions on swimming speed and efficiency one makes by neglecting hydrodynamic interactions are never quite right. The additive approximation (estimating the resistance of the assembly by adding the contributions of the parts, each computed in the absence of the other parts) and, in particular, RFT miss completely the existence of optimal values for the flagellar length that maximize energetic or propulsion efficiency and lead to wrong predictions for the flagellar length, giving maximal swimming speed.

There is, however, a way to rescue the valuable intuitive idea of adding the contributions of the parts to estimate the resistance of the whole: the individual contributions must be evaluated *in the presence of the other body parts*. By carefully exploiting this notion of additivity (which rests on the linearity of the Stokes system), rather than adding the resistance matrix of the individual components (which are computed by assuming that each individual part is alone in free space), one can obtain simple corrections (a single scalar correcting factor for the swimming speed in the case of a spherical body powered by a rotating helical flagellum), so that the predictions on the swimming speed and efficiency of a microswimmer can be obtained by using simplified hydrodynamic models, such as RFT.

Optimal design problems become approachable in this way, as shown in our results. In this study, the problem of finding the optimal length for a helical flagellum of given shape, pushing a spherical head of fixed size and powered by a rotary motor of given speed, is formulated and solved for several different performance measures. Clearly, extensions to more complex swimmers is desirable and interesting, starting from conceptual models such as those in which the tail consists of Purcell's three-link swimmer, or else is a chain of *N* identical units and one is interested in the optimal *N*, and up to concrete devices in the context of microrobotics. We believe that our analysis can provide the starting point for such extensions, although it is clear that optimal design of more complex swimmers than the one analyzed in this study will require devising new and likely more complex “correction” strategies than one based on a single scalar parameter.
